# Clomifene citrate or unstimulated intrauterine insemination compared with expectant management for unexplained infertility: pragmatic randomised controlled trial 

**DOI:** 10.1136/bmj.a716

**Published:** 2008-08-07

**Authors:** S Bhattacharya, K Harrild, J Mollison, S Wordsworth, C Tay, A Harrold, D McQueen, H Lyall, L Johnston, J Burrage, S Grossett, H Walton, J Lynch, A Johnstone, S Kini, A Raja, A Templeton

**Affiliations:** 1 Department of Obstetrics and Gynaecology, University of Aberdeen, Aberdeen AB25 2ZD; 2 Centre for Statistics in Medicine, University of Oxford, Wolfson College Annexe, Oxford; 3 Health Economics Research Centre, University of Oxford, Oxford; 4 Simpson Centre for Reproductive Health, Royal Infirmary of Edinburgh, Edinburgh EH16 4SA; 5 Assisted Conception Unit, Ninewells Hospital, Dundee; 6 Falkirk and District Royal Infirmary, Falkirk; 7 ACS Unit, Ground Floor, Walton Building, Royal Infirmary, Glasgow

## Abstract

**Objective** To compare the effectiveness of clomifene citrate and unstimulated intrauterine insemination with expectant management for the treatment of unexplained infertility.

**Design** Three arm parallel group, pragmatic randomised controlled trial.

**Setting** Four teaching hospitals and a district general hospital in Scotland.

**Participants** Couples with infertility for over two years, confirmed ovulation, patent fallopian tubes, and motile sperm.

**Intervention** Expectant management, oral clomifene citrate, and unstimulated intrauterine insemination.

**Main outcome measures** The primary outcome was live birth. Secondary outcome measures included clinical pregnancy, multiple pregnancy, miscarriage, and acceptability.

**Results** 580 women were randomised to expectant management (n=193), oral clomifene citrate (n=194), or unstimulated intrauterine insemination (n=193) for six months. The three randomised groups were comparable in terms of age, body mass index, duration of infertility, sperm concentration, and motility. Live birth rates were 32/193 (17%), 26/192 (14%), and 43/191 (23%), respectively. Compared with expectant management, the odds ratio for a live birth was 0.79 (95% confidence interval 0.45 to 1.38) after clomifene citrate and 1.46 (0.88 to 2.43) after unstimulated intrauterine insemination. More women randomised to clomifene citrate (159/170, 94%) and unstimulated intrauterine insemination (155/162, 96%) found the process of treatment acceptable than those randomised to expectant management (123/153, 80%) (P=0.001 and P<0.001, respectively).

**Conclusion** In couples with unexplained infertility existing treatments such as empirical clomifene and unstimulated intrauterine insemination are unlikely to offer superior live birth rates compared with expectant management.

**Trial registration** ISRCT No: 71762042

## Introduction

Infertility affects one in seven couples^1^ and is described as unexplained when standard investigations (semen analysis, tubal patency tests, and assessment of ovulation) fail to find any abnormalities. Unexplained infertility affects a quarter of infertile couples,^1^ some of whom have a reasonable chance of spontaneous live birth.^2^ Expectant management, however, is not a popular option, and several empirical interventions have been used for many years without rigorous prior evaluation. Commonly used first line treatments for unexplained infertility include clomifene citrate and intrauterine insemination.^3^ ^4^ ^5^

Oral clomifene citrate, a popular choice for the treatment of unexplained infertility, is inexpensive, requires little clinical monitoring, and is thought to correct subtle ovulatory dysfunction.^3^ Concerns about multiple pregnancies induced by clomifene and a potential risk of ovarian cancer, however, underline the need to weigh the risks and benefits.^5^ ^6^ ^7^

Intrauterine insemination of sperm can potentially enhance pregnancy rates by helping to overcome the cervical barrier. Observational data suggest a threefold increase in pregnancy rates with intrauterine insemination alone, with a further increase with concomitant ovarian stimulation with gonadotrophin (superovulation).^8^

Superovulation with intrauterine insemination is superior to unstimulated intrauterine insemination,^9^ but gonadotrophins can cause ovarian hyperstimulation and multiple pregnancy.^10^ ^11^ A recent trial failed to show any advantage of superovulation with intrauterine insemination over expectant management in selected couples with a good chance of spontaneous pregnancy^12^ and Goverde et al failed to show any superiority over unstimulated intrauterine insemination,^13^ but there are no adequately designed trials of unstimulated intrauterine insemination versus expectant management. The current recommendation in the United Kingdom favours the use of unstimulated intrauterine insemination in unexplained infertility.^7^

Systematic reviews have exposed the uneven quality in methods of primary studies and the uncertainties of the available evidence.^3^ ^4^ ^7^ ^8^ ^14^ They have highlighted the need for a definitive trial with adequate power to assess the effectiveness of clomifene citrate and unstimulated intrauterine insemination in unexplained subfertility.

We compared the clinical effectiveness and cost of these commonly used treatments against expectant management. We focus here on clinical outcomes.

## Methods

### Study design

The study was a three arm, parallel group, pragmatic randomised controlled trial. We recruited patients from four major teaching hospitals and one district general hospital in Scotland. Inclusion criteria were at least two years of infertility, bilateral tubal patency (demonstrated by laparoscopy or hysterosalpingography), ovulation demonstrated by appropriately timed mid-luteal progesterone, and normal semen variables (according to World Health Organization criteria). We also included coupleswith minimum sperm motility of 20% or minimal endometriosis (rAFS stage 1).

*Expectant management*—This involved six months during which no clinic visits or medical interventions were scheduled. Couples were given general advice regarding the need for regular intercourse, but no specific measures such as basal temperature charts or luteinising hormone kits were recommended.

*Clomifene citrate*—Women randomised to clomifene citrate received an oral dose of 50 mg between day two and six of each treatment cycle. During the first cycle they underwent a transvaginal scan and mid-luteal progesterone check. Subsequent cycles were monitored by a mid-luteal progesterone check alone. Couples were advised to have intercourse on days 12-18 of the cycle. If three or more ovarian follicles were detected by scan in the first cycle, the cycle was cancelled and the couple advised to avoid intercourse. In the next cycle, women who were overstimulated on the first cyclestarted on a reduced dose of clomifene (25 mg) and were monitored in the same way as they would be for a first cycle (that is, scan on day 12 and blood test for progesterone on day 21) with a further reduction to alternate days of 25 mg offered in the next cycle if necessary. As women were known to be ovulatory, we did not anticipate progesterone concentrations to indicate non-ovulation. A single low mid-luteal progesterone concentration (suggestive of anovulation) could be attributed to a mistimed blood test and, given the pragmatic nature of the trial, did not result in a change of protocol. In the event of low concentrations in successive cycles, we intended to treat the women as anovulatory using the standard clinic protocol of incremental doses of clomifene.

*Intrauterine insemination*—Women were asked to monitor mid-morning urinary luteinising hormone concentrations from day 12 of their cycle using Clearview (Unipath, Bedford). A single insemination was performed 20-30 hours after an endogenous surge was detected. Semen was prepared with a swim-up technique with Puresperm (Nidacon, Hunter Scientific, Saffron Walden, Essex) density gradient followed by resuspension in a sperm buffer. A maximum of 0.5 ml suspension of processed spermatozoa was introduced into the uterine cavity through the cervix with a 10 cm intrauterine insemination catheter. Couples were advised to avoid intercourse from day 12 of the cycle until the day of insemination. A woman who missed a luteinising hormone surge did not receive intrauterine insemination in that cycle but continued to monitor luteinising hormone concentrations in the next cycle, with a view to insemination as originally planned.

### Randomisation and follow-up

An independent statistician generated the randomisation allocation sequence. Research nurses enrolled participants at each centre and assigned them to their groups using a central telephone randomisation system based in Aberdeen (the coordinating centre). The minimisation algorithm balanced allocation of treatment by maternal age, parity, and duration of subfertility. Women were stratified by centre, and blinding was not possible because of the nature of the interventions. The duration of intervention was six months. All five recruiting centres followed standardised written treatment protocols relating to drug doses, counselling before treatment, ultrasound scans, and laboratory tests. Information sheets for patients relating to treatment or timing of intercourse were also similar.

Women were followed up for six months after randomisation. A pregnancy test was performed two weeks after intrauterine insemination or (in the absence of a period) by day 28 in the other groups. Clinical pregnancy was confirmed by the presence of an intrauterine gestational sac on ultrasonography, with a fetal heartbeat five weeks later. Women who became pregnant but later miscarried within six months of randomisation were allowed to have further treatment in their randomised groups for the rest of their allocated time. We included spontaneous pregnancies in the clomifene citrate and intrauterine insemination arms in the final analysis.

### Outcomes

The primary outcome was live birth per woman. Secondary outcomes included clinical pregnancy rate per woman, multiple pregnancy rate, acceptability, adverse events, anxiety, and depression.

Research nurses completed structured records of clinical and procedural details. We used questionnaires completed by women at randomisation and at six months to collect data on acceptability of treatment, side effects, anxiety, and depression. Anxiety and depression were measured with the hospital anxiety and depression scale, with scores of 11 or more suggestive of the relevant mood disorder.^15^ Women were asked to describe how acceptable their treatment was with respect to the process and outcome of their treatment separately by using Likert items with six possible responses, ranging from “totally acceptable” to “totally unacceptable.” Women who were pregnant at six months after randomisation were followed up to obtain data on delivery.

### Statistical analysis

We designed this trial to test two primary comparisons: expectant management versus clomifene citrate and expectant management versus unstimulated intrauterine insemination. We aimed to recruit 190 couples in each group (1:1:1)—a total of 570 couples (including an allowance for a 10% loss to follow-up).

We estimated that six months of expectant treatment would be associated with a live birth rate of 10%.^2^ Based on the pooled odds ratio of 2.4 in favour of clomifene reported in the Cochrane review by Hughes et al^14^ at the time the study was designed in 2001, we envisaged that we needed 168 women in each group to show a clinically meaningful improvement in live birth outcomes (from 10% to 22%; odds ratio 2.5) for clomifene versus expectant management, with 80% power at the 5% level of significance. Our proposed sample size would allow detection of a similar difference between expectant management and unstimulated intrauterine insemination (10% to 22%; odds ratio 2.5). In a randomised trial of unstimulated versus stimulated intrauterine insemination versus in vitro fertilisation by Goverde et al,^13^ the cumulative live birth rate in couples undergoing unstimulated intrauterine insemination was 31%. Taking these data into account, along with the invasiveness of the treatment and the perceived benefit to couples, we expected to have in excess of 95% power to detect a difference in live birth rates of 20% (10% to 30%; odds ratio 4) between expectant management and unstimulated intrauterine insemination .

If both active treatments (clomifene citrate and unstimulated intrauterine insemination) were found to be more effective than expectant management, we anticipated having over 85% power at the 5% level of significance to detect an absolute difference of 15% (15% to 30%) in live birth rates between the active treatment groups.

Data were entered on a computer held database and analysed with SPSS for Windows (SPSS, Chicago, IL; version 14). The analysis strategy was defined a priori and addressed the clinically relevant comparisons. Our primary aim was to compare clomifene citrate versus expectant management and unstimulated intrauterine insemination versus expectant management. We intended to compare the two active treatments only if both were found to be superior to expectant management. We decided not to conduct a global test across all three groups as the direct comparisons were of clinical relevance.^16^ We used intention to treat analyses throughout, with a secondary per protocol analysis conducted on live birth rates only. We analysed categorical variables with χ^2^ tests, used logistic regression models to adjust for maternal age, parity, duration of infertility, and recruitment centre, and calculated odds ratios with confidence intervals (95% for the primary outcome, 99% for secondary outcomes). On the basis of the crude live birth rates and 95% confidence intervals for the absolute risk reductions calculated with Newcombe’s method for two unpaired proportions,^17^ we computed the numbers needed to treat for benefit (NNB) or harm (NNH) as appropriate, along with their 95% confidence intervals. We initially compared time to pregnancy leading to a live birth with log rank tests, then used Cox proportional hazards models to make adjustments. Interaction tests for subgroup analyses (planned a priori) were obtained with logistic regression modelling live birth per woman. Responses to the questions regarding the acceptability of treatment were grouped into “acceptable” or “unacceptable” for the purposes of analysis. A P value of 0.05 was considered significant for the primary outcome, while a value of 0.01 was used for all secondary outcomes.

## Results

A total of 580 couples were recruited between September 2001 and September 2005 (fig 1).

**Figure fig1:**
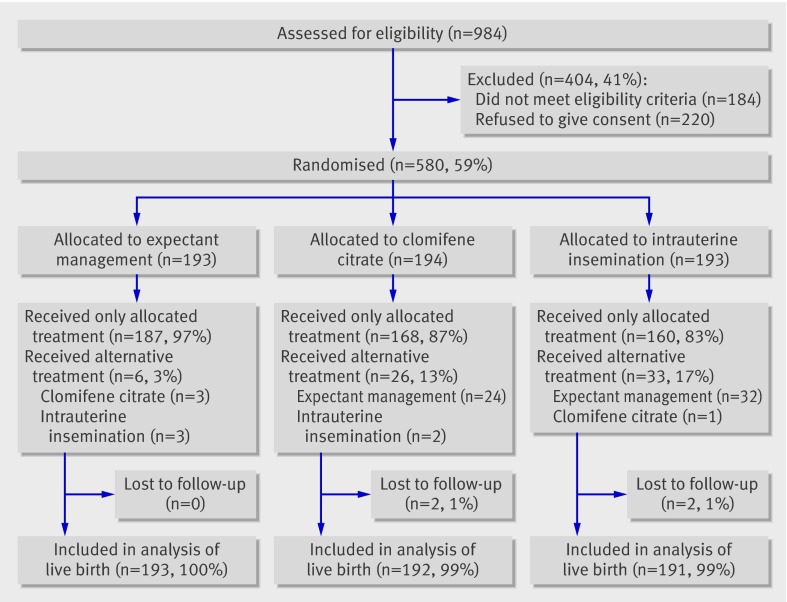
**Fig 1** Trial flow chart

### Baseline characteristics

The three groups (expectant management, clomifene citrate, and intrauterine insemination) were comparable in terms of women’s age, men’s age, BMI, semen variables, and proportion of couples with primary infertility (table 1).[Table tbl1] The median duration of infertility was 30 months in each of the randomised groups and only a few individuals (under 10%) had endometriosis or mild male factor infertility problems.

**Table 1 tbl1:** Baseline characteristics at randomisation according to allocation to expectant management, clomifene citrate, or unstimulated intrauterine insemination for unexplained infertility. Figures are numbers (percentages) unless stated otherwise

	Expectant management (n=193)	Clomifene citrate (n=194)	Insemination (n=193)
Mean (SD) age (years):			
Women	32 (3.4)	32 (3.5)	32 (3.7)
Men	34 (5.1)	34 (5.1)	34 (5.2)
Median (IQR) duration of infertility (months)	30 (25-38)	30 (24-38)	30 (25-40)
Primary infertility	135 (70)	144 (74)	134 (69)
Mild endometriosis,	17 (9)	9 (5)	13 (7)
Surgical treatment for endometriosis*	3 (18)	2 (22)	1 (8)
Mild male factor infertility	9 (5)	11 (6)	14 (7)
Median (IQR) BMI (women)	23 (21-25)	23 (22-26)	23 (21-26)
Sperm variables:			
Median (IQR) density (million/ml)	62 (39-95)	65 (38-105)	58 (35-98)
Mean (SD) motility %	52 (15.6)	53 (16.4)	53 (15.6)
HADS subscale ≥11:			
Anxiety	29 (15)	28 (14)	23 (12)
Depression	3 (2)	1 (1)	2 (1)

IQR=interquartile range; BMI=body mass index; HADS=hospital anxiety and depression scale.

*Percentage of those who had mild endometriosis.

### Active treatments

Of the 194 women randomised to clomifene citrate, 93 (48%) received six completed cycles of clomifene citrate and 18 (9%) received none over the six month trial period. During the trial period, 37/193 (19%) women randomised to unstimulated intrauterine insemination received six completed cycles of intrauterine insemination and 26 (13%) received none. The median (interquartile range) number of treatment cycles received was 5 (2-6) for the clomifene citrate group and 4 (2-5) for the intrauterine insemination group.

Of the 883 cycles where the intention was for the women to receive clomifene, 31 (4%) cycles in 20 women resulted in an exaggerated response (three or more follicles). There were 785 cycles where the intention was for the women to receive intrauterine insemination; 25 (3%) of these cycles resulted in missing the luteinising hormone surge and 13 women missed the surge at least once.

### Live birth

Live birth rates in the three randomised groups were 32/193 (17%) for expectant management, 26/192 (14%) for clomifene citrate, and 43/191 (23%) for unstimulated intrauterine insemination (table 2). Three women (2%) in the clomifene citrate group and 14 (7%) in the intrauterine insemination group became pregnant spontaneously and had a live birth. Compared with expectant management, the odds ratio of a live birth was 0.79 (95% confidence interval 0.45 to 1.38) with clomifene citrate and 1.46 (0.88 to 2.43) with unstimulated intrauterine insemination. Adjustment for maternal age, parity, duration of infertility, and recruitment centre gave similar results (table 2), as did the per protocol analysis.[Table tbl2]

**Table 2 tbl2:** Analysis of live birth for expectant management compared with clomifene citrate or unstimulated intrauterine insemination

Analysis	No (%) in expectant management group	Clomifene citrate *v* expectant management				Intrauterine insemination *v* expectant management		
		No (%)	OR (95% CI), P value			No (%)	OR (95% CI), P value	
			Crude	Adjusted*			Crude	Adjusted*
Intention to treat	32/193 (17)	26/192 (14)	0.79 (0.45 to 1.38), 0.49	0.80 (0.45 to 1.42), 0.45		43/191 (23)	1.46 (0.88 to 2.43), 0.18	1.53 (0.91 to 2.56), 0.11
Per protocol	30/187 (16)	24/175 (14)	0.83 (0.47 to 1.45), 0.64	0.83 (0.46 to 1.50), 0.53		31/167 (19)	1.19 (0.69 to 2.07), 0.63	1.25 (0.71 to 2.18), 0.44

*Adjusted for maternal age, parity, duration of infertility, and recruitment centre.

The number needed to treat for harm with clomifene citrate was 33 (95% confidence interval NNH 10 to ∞ to NNB 24). This indicates that if 33 women were treated with clomifene citrate, one fewer would have a live birth than if they all received expectant management, with the confidence interval indicating the uncertainty of the estimate. The number needed to treat for benefit with unstimulated intrauterine insemination was 17 (NNH 51 to ∞ to NNB 7). Therefore, 17 women would need to undergo intrauterine insemination rather than expectant management for one additional woman to have a live birth, with the range of likely values for this estimate given by the confidence interval.

There were no significant differences in the time to pregnancy leading to a live birth with clomifene citrate (P=0.41) or unstimulated intrauterine insemination (P=0.17) compared with expectant management (fig 2). Compared with expectant management, the adjusted hazard ratio for the time to a pregnancy leading to a live birth was 0.83 (99% confidence interval 0.42 to 1.63) for clomifene citrate and 1.40 (0.77 to 2.56) for unstimulated intrauterine insemination.

**Figure fig2:**
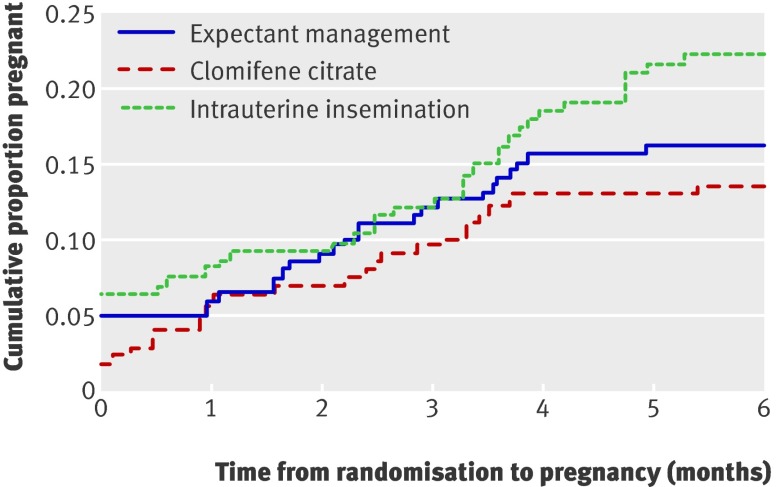
**Fig 2** Time to pregnancy leading to live birth in groups allocated to clomifene citrate, expectant management, or unstimulated intrauterine insemination. For women with live birth, time to event was defined as number of months between randomisation and estimated date of last menstrual period; women without live birth were censored at end of their follow-up

The subgroup analyses for live birth per woman found no modifiers of the effects of clomifene citrate or unstimulated intrauterine insemination compared with expectant management (table 3). The treatment effects were similar between couples with and without pure unexplained infertility, between couples with and without mild male factor infertility, and between couples with primary and secondary infertility. The number of live births in each subgroup and treatment combination, however, was small.

**Table 3 tbl3:** Subgroup analysis of live birth by diagnostic group for expectant management compared with clomifene citrate or unstimulated intrauterine insemination, with crude and adjusted P values*

	No (%) in expectant management group	Clomifene citrate *v* expectant management				Intrauterine insemination *v* expectant management		
		No (%)	Crude	Adjusted†		No (%)	Crude	Adjusted†
All	32/193 (17)	26/192 (14)	—	—		43/191 (23)	—	—
Pure unexplained infertility:								
No	6/26 (23)	3/19 (16)	0.73	0.76		5/26 (19)	0.33	0.28
Yes	26/167 (16)	23/173 (13)				38/165 (23)		
Mild male infertility factor:								
No	30/184 (16)	23/181 (13)	0.61	0.53		41/177 (23)	0.39	0.25
Yes	2/9 (22)	3/11 (27)				2/14 (14)		
Mild endometriosis:								
No	28/176 (16)	26/183 (14)	—	—		40/178 (22)	0.62	0.68
Yes	4/17 (24)	0/9				3/13 (23)		
Mild male infertility factor and mild endometriosis:								
No	32/193 (17)	26/191 (14)	—	—		43/190 (23)	—	—
Yes	0/0	0/1				0/1		
Type of infertility:								
Primary	24/135 (18)	19/142 (13)	0.59	0.47		29/132 (22)	0.49	0.39
Secondary	8/58 (14)	7/50 (14)				14/59 (24)		

*For treatment*subgroup interaction term.

†Adjusted for maternal age, parity, duration of infertility, and recruitment centre.

### Secondary outcomes

Table 4 shows other clinical outcomes in the three randomised groups.[Table tbl4] Clinical pregnancy rates were comparable between expectant management and clomifene citrate (17% *v* 15%) and expectant management and unstimulated intrauterine insemination (17% *v* 23%). Rates of miscarriage and ectopic pregnancy were also similar in the two comparisons, as were rates of multiple pregnancy.

Side effects including abdominal pain, bloating, hot flushes, nausea, and headaches were more common in the clomifene citrate group than in the other groups, affecting about 10-20% of women. Despite this, women on active treatments found the process of treatment more acceptable than those randomised to expectant management. The proportion of women scoring 11 or more on the anxiety or depression subscales at six months was similar across the three groups.

**Table 4 tbl4:** Analysis of secondary outcomes for expectant management compared with clomifene citrate or unstimulated intrauterine insemination

	No (%) in expectant management group	Clomifene citrate *v* expectant management				Intrauterine insemination *v* expectant management		
		No (%)	OR (99% CI), P value			No (%)	OR (99% CI), P value	
			Crude	Adjusted*			Crude	Adjusted*
Clinical pregnancy per woman†	33/193 (17)	29/192 (15)	0.86 (0.42 to 1.76), 0.69	0.88 (0.42 to 1.82), 0.64		43/191 (23)	1.41 (0.73 to 2.74), 0.23	1.47 (0.75 to 2.89), 0.14
Multiple clinical pregnancy per woman‡	2/193 (1)	2/192 (1)	1.01 (0.08 to 13.39), 1.00	—		1/191 (1)	0.50 (0.02 to 11.92), 1.00	—
Miscarriage¶	14/46 (30)	10/38 (26)	0.82 (0.23 to 2.87), 0.86	—		9/55 (16)	0.45 (0.13 to 1.56), 0.15	0.35 (0.09 to 1.40), 0.05
Ectopic pregnancy¶	1/46 (2)	0/38 (0)	P=1.00	—		2/55 (4)	1.70 (0.07 to 41.56), 1.00	—
Preterm birth§	5/31 (16)	3/26 (12)	0.68 (0.09 to 5.12), 0.72	—		6/43 (14)	0.84 (0.16 to 4.59), 1.00	—
Treatment related hospital admission	2/160 (1)	2/168 (1)	0.95 (0.07 to 12.71), 1.00	—		0/163 (0)	P=0.25	—
Abdominal pain	5/159 (3)	40/169 (24)	9.55 (2.71 to 33.67), <0.001	9.76 (2.75 to 34.65), <0.001		12/164 (7)	2.43 (0.60 to 9.88), 0.15	—
Vaginal bleeding	4/159 (3)	7/165 (4)	1.72 (0.33 to 8.86), 0.58	1.65 (0.32 to 8.66), 0.44		10/164 (6)	2.52 (0.53 to 11.88), 0.19	—
Nausea	4/159 (3)	22/166 (13)	5.92 (1.42 to 24.78), 0.001	6.14 (1.43 to 26.44), 0.001		3/164 (2)	0.72 (0.10 to 5.28), 0.72	—
Vomiting	0/158 (0)	1/165 (1)	P=1.00	—		0/164 (0)	—	—
Headache	6/159 (4)	33/167 (20)	6.28 (1.92 to 20.50), <0.001	6.43 (1.95 to 21.25), <0.001		4/164 (2)	0.64 (0.12 to 3.45), 0.54	—
Hot flushes	4/159 (3)	30/170 (18)	8.30 (2.04 to 33.79), <0.001	8.63 (2.10 to 35.49), <0.001		0/164 (0)	—	—
Bloating	0/158 (0)	33/168 (20)	P<0.001	—		6/164 (4)	—	—
Process of treatment acceptable	123/153 (80)	159/170 (94)	3.53 (1.35 to 9.20), 0.001	3.77 (1.42 to 9.99), <0.001		155/162 (96)	5.40 (1.75 to 16.64), <0.001	5.55 (1.78 to 17.25), <0.001
Outcome of treatment acceptable	82/148 (55)	100/168 (60)	1.18 (0.66 to 2.13), 0.53	1.20 (0.66 to 2.17), 0.44		117/159 (74)	2.24 (1.20 to 4.21), 0.001	2.27 (1.19 to 4.31), 0.001
HADS subscale ≥11:								
Anxiety	31/171 (18)	34/175 (19)	1.09 (0.54 to 2.21), 0.86	1.09 (0.53 to 2.25), 0.75		22/173 (13)	0.66 (0.30 to 1.43), 0.22	0.64 (0.29 to 1.42), 0.15
Depression	4/170 (2)	4/174 (2)	0.98 (0.16 to 6.17), 1.00	—		2/172 (1)	0.49 (0.05 to 4.63), 0.45	—

HADS=hospital anxiety and depression scale.

*Adjusted for maternal age, parity, duration of infertility, and recruitment centre.

†At least one fetal heart beat seen on ultrasound.

‡At least two fetal heart beats seen on ultrasound.

¶Denominator is biochemical pregnancy (positive pregnancy test).

§Gestation period ≤37 weeks; denominator is live births with known gestation period.

## Discussion

Clomifene citrate or unstimulated intrauterine insemination seems to be no more effective than expectant management in couples with unexplained infertility.

### Strengths and weaknesses

We compared the effectiveness of both clomifene citrate and unstimulated intrauterine insemination versus expectant management. The inclusion of a control group (expectant management) means that our results reflect the true marginal effect of these popular interventions. The trial is strengthened by its pragmatic, multicentre approach and the choice of live birth per woman as its end point. We exceeded our recruitment target with less than 1% loss to follow-up; in addition, the number of women in the expectant management and clomifene citrate arms exceeded the number randomised in all of the previous clomifene citrate trials pooled together.^14^

The inclusion of cases of mild male factor infertility and minimal endometriosis might have introduced an element of clinical heterogeneity. The presence of these cases in this pragmatic trial, however, can be justified on the grounds that they are managed in the same way as “true” unexplained infertility.^6^ Although there were few live births within each subgroup, subgroup analyses indicate similar treatment effects in these groups. Our choice of clinical protocols for the intervention arms reflects current practice in Scotland and the rest of the UK,^7^ but the results might not be generalisable to other populations and alternative drug regimens. In particular, this trial does not address the issue of a combined approach with clomifene citrate and intrauterine insemination, which should be the focus of future trials.

### Interpretation within context of setting and intervention

At the time this trial was conducted, the Cochrane review on clomifene citrate in unexplained infertility suggested that it had a beneficial effect and supported its use on grounds of low cost and ease of administration.^14^ Fertility guidelines in the UK and the United States endorsed this view^5^ ^7^ but highlighted the need for a definitive trial. While both 100 mg and 50 mg of clomifene citrate have been used in previous trials, our decision to use the lower dose in this cohort of ovulatory women was guided by concerns about the risk of high order multiple pregnancy.^18^ The rationale for ovarian stimulation in women with documented ovulation has been to correct subtle disorders of ovulation not identified by conventional tests and to increase the number of oocytes per cycle.^3^ Yet, as an agonist-antagonist at the level of the oestrogen receptor, antioestrogenic effects of clomifene citrate on the endometrium, uterine blood flow, and quality of cervical mucus could affect its ability to enhance pregnancy rates.^19^ A recent placebo controlled trial with 140 women with unexplained infertility failed to show higher rates of live birth after treatment with 100 mg clomifene citrate.^20^

Concern about iatrogenic multiple pregnancies has influenced a national guideline in the UK to advocate the use of unstimulated intrauterine insemination.^7^ A Cochrane review showed the rate of live birth to be higher with stimulated intrauterine insemination but found no suitable trials comparing unstimulated intrauterine insemination with expectant management.^21^ The only relevant trial in the literature used intrauterine insemination cycles as the unit of analysis and a crossover design but did not provide outcome data from before the crossover.^22^ A recent trial showed that superovulation with intrauterine insemination offered no advantage in a subgroup of couples with unexplained infertility with a good chance of spontaneous pregnancy.^12^ Recruited couples, however, had a shorter duration of infertility than in our trial and hence a higher expectation of spontaneous pregnancy.^2^ In contrast, our trial essentially compares both first line treatments (clomifene citrate and unstimulated intrauterine insemination) for unexplained infertility in an unselected population with mixed prognosis and includes clinical and cost effectiveness as outcomes. It was previously believed that technical and procedural aspects of intrauterine insemination could influence the outcome of treatment. A systematic review has failed to confirm the superiority of double insemination over single insemination (as performed in this trial).^23^ Fewer than 20% of couples randomised to unstimulated intrauterine insemination received six cycles of treatment over the six month trial period. It has been noted in a previous trial that not all couples randomised to intrauterine insemination treatment receive their full quota of six cycles within this time period.^13^ We cannot exclude the possibility that exposure to more cycles of intrauterine insemination could have led to a higher live birth rate, but for practical reasons we needed to stipulate a time dependent exposure to the trial intervention.

Our results also show that women with infertility are reassured by active treatment and are less satisfied with an expectant approach. Similar concerns in the past have limited the number of fertility trials with a control arm.^2^ Comparable anxiety and depression scores in the randomised groups suggest that preference for active treatment did not translate into greater mental wellbeing in those randomised to clomifene citrate or unstimulated intrauterine insemination. As both active treatments have resource implications, information on the costs of the two treatments is important to consider and will be reported elsewhere.

Interpretation of our findings needs to take into account the population sampled. We are unable to provide demographic information on those women who did not agree to participate in this trial as we had no ethical approval to collect such data. Criteria for the diagnosis of “true” unexplained infertility are still debatable.^24^ Accurate diagnosis is influenced by the nature, number, and precision of the tests used. The present trend towards a less invasive approach to tubal patency testing^7^ ^12^ is supported by data showing that additional laparoscopy after hysterosalpingography does not enhance the success of unstimulated intrauterine insemination.^25^ Without routine laparoscopy, however, it is impossible to exclude the possibility of endometriosis.

### Statistical approach

An approach commonly adopted in multi-arm trials involves undertaking one global test across all treatment groups. Such tests do not identify which treatments are different and can have limited power to detect genuine differences.^26^ We designed our trial to make direct comparisons with a control (expectant management); therefore, there was a logical basis for performing separate significant tests for each active treatment versus expectant management.^16^ ^26^ Knowing which of the active treatments is the more effective is clinically relevant only if both treatments prove to be superior to expectant management.

As recommended and planned a priori, we adjusted our results for the minimisation and stratification factors used in the randomisation scheme^27^ but have also presented crude results so that the impact of these adjustments can be assessed. As some women did not receive their randomised treatment, we presented a per protocol analysis of live birth rates, along with the intention to treat analysis, to investigate what effects this might have on our conclusions. We found that unstimulated intrauterine insemination did not enhance live birth, though the upper limit of the 95% confidence interval for the number needed to treat to benefit was 7, a figure that might be perceived to be clinically worthwhile.

### Conclusions

Spontaneous live birth rates in a randomised cohort of women with unexplained infertility do not seem to be enhanced by common first line treatments—clomifene citrate or unstimulated intrauterine insemination. These results challenge current practice, as endorsed by a national guideline in the UK.^7^

**What is already known on this topic**

Infertility is unexplained in a quarter of all couples with fertility problems
Clomifene citrate or unstimulated intrauterine insemination, commonly used to treat such couples, are endorsed in the UK by the NICE fertility guideline
Previous evidence based on a number of small randomised trials supports the use of clomifene in unexplained subfertility

**What this study adds**

Clomifene citrate or unstimulated intrauterine insemination are unlikely to be more effective than no treatment (expectant management)

## Notes

We thank all the women who participated in the trial. Elaine Stirton collated data from the recruiting centres and provided secretarial support to the trial team, and Sarah McSwiney formatted the document. M Hamilton (Aberdeen), S Irvine, R Anderson, C Duncan, C West (Edinburgh), G P Cumming (Elgin), R Yates (Glasgow), L Caird and A Giwa-Osagie (Inverness), H Currie (Dumfries), and J Gemmell (Paisley) referred patients for recruitment.

Contributors: SB was the principal investigator, designed the trial, led the funding application, wrote the protocol, managed the project, recruited participants, interpreted the data and results, and wrote the first draft of the paper. JM advised on the study design and data analysis and helped to draft the paper. KH helped to design and performed the statistical analysis, interpreted the data and results, and helped to draft the paper. SW designed the health economic methods, performed the cost analysis, interpreted the results, and helped to draft the paper. LJ recruited patients and entered data. JB, SG, JL, HW, AJ, SK, and AR recruited patients and collected data. AH, HL, DMcQ, CT, and AT all contributed towards the initiation of the project, trial design, recruitment of patients, and interpretation of results. SB is guarantor.

Ethical approval: Multicentre research ethics committee, Scotland.

Provenance and peer review: Not commissioned; externally peer reviewed.

## Notes

**Cite this as:** *BMJ* 2008;337:a716
